# Taming the wild: domesticating untapped northern fruit tree and shrub resources in the era of high-throughput technologies

**DOI:** 10.1093/aobpla/plae074

**Published:** 2025-01-04

**Authors:** Jérôme Gélinas Bélanger

**Affiliations:** Department of Plant Science, McGill University, Macdonald Campus, 21111 Rue Lakeshore, Ste-Anne-de-Bellevue, H9X 3V9, Québec, Canada; Centre de recherche sur les grains (CÉROM) Inc., 740 Chem. Trudeau, Saint-Mathieu-de-Beloeil, J3G 0E2, Québec, Canada

**Keywords:** domestication, fruit crops, northern agroecosystems, breeding, genome editing, early flowering

## Abstract

New crop`s need to emerge to provide sustainable solutions to climate change and increasing abiotic and biotic constraints on agriculture. A large breadth of northern fruit trees and shrubs exhibit a high potential for domestication; however, obstacles to implementing traditional breeding methods have hampered or dissuaded efforts for improvement. This review article proposes a unique roadmap for *de novo* domestication of northern fruit crops, with a focus on biotechnological (e.g. genome editing, rapid cycle breeding, and *in planta* transformation) approaches that can boast rapid evolutionary gains. In addition, numerous biotechnological (e.g. virus-induced flowering and grafting-mediated flowering) and breeding strategies (e.g. adaptation of speed breeding to fruit trees) that can hasten the transition from juvenility to sexual maturity are described. A description of an accelerated genetic breeding strategy with insights for 16 underutilized species (e.g. shagbark hickory, running serviceberry, horse chestnut, and black walnut) is provided to support their enhancement. Deemed unrealistic only a decade ago, progress in the realm of bioengineering heralds a future for northern orphan crops through the implementation of fast-tracked crop improvement programs. As such, the roadmap presented in this article paves the way to integrating these novel biotechnological discoveries and propel the development of these forgotten crops in a sustainable and timely manner.

## Teaser

To improve food security and limit the pervasive effects of climate change, new crops need to be developed and domesticated in a timely manner. In this article, a breeding pipeline relying on many biotechnological (e.g. in planta transformation, genome editing, and virus-induced flowering) and conventional (e.g. modification of growth conditions) avenues is proposed to domesticate orphan northern fruit trees, with a focus on techniques that can hasten the transition from juvenility to sexual maturity. To support the proposed roadmap, crop improvement avenues for 16 underutilized species (e.g. shagbark hickory, running serviceberry, horse chestnut, and black walnut) are proposed and detailed. Overall, the strategy presented in this article heralds a future for northern orphan crops through the implementation of fast-tracked crop improvement programs.

## Introduction

The domestication of fruit crops from the wild is a long-term process involving multiple technical challenges and a large degree of uncertainty. Fruit trees typically have a long juvenile period that delays the selection process and a large size which hinders large-scale breeding programs (Hanke *et al.* 2007). Although several northern fruit tree crops exhibit tremendous potential for the production of highly nutritious and commercially viable fruits, their domestication process has (in the best cases) stagnated or been deemed too difficult and thus never been initiated. Nonetheless, recent progress in fruit tree research (e.g. rapid cycle breeding approach, virus-induced flowering [VIF], *in vivo* callus regeneration along with *in planta* transformation techniques, and genome editing) offers multiple avenues for the development of fast-tracked streamlined genetic improvement procedures for these hard-to-domesticate species. The main purpose of this review is to highlight the current domestication toolkit available to breeders and scientists as a means to propel the cultivation of novel northern fruit tree crops. To do so, a streamlined four-step domestication roadmap to improve fruit crops displaying a high commercial potential is proposed and innovative techniques that can be used to kickstart breeding programs specifically dedicated to these niche crops are described. Given the limited sources of funding for such endeavours, this pipeline was built with the intent of obtaining rapid gains (i.e. quick return on investment) and fostering commercial success. To the best of our knowledge, no review article with a specific focus on undomesticated northern fruit crops is currently available in the literature although a few have addressed the same topic but only for tropical species (Akinnifesi *et al.* 2007; Leakey and Akinnifesi 2008; Huang 2022). Still, these articles include little to no information regarding the role of novel bioengineering technologies in this *de novo* domestication process. As a whole, the objectives of this article are to (i) review novel domestication technologies that can achieve quick gains in uncultivated fruit and nut tree species; (ii) propose a crop improvement pipeline to support a streamlined *de novo* domestication process; and (iii) highlight the potential for crop development of various wild northern fruit and nut species.

## The roadmap to domestication

By definition, domestication traits are phenotypes associated with the initial formation of the new cultivated form of species from their wild ancestors (Meyer and Purugganan 2013). Throughout history, the biggest driver of human-driven crop evolution has been the stacking of loss-of-function alleles in domestication genes (Luo *et al.* 2022). In crops, loss-of-function point mutations are the most common variants found in domestication genes, with a large proportion of the lesions causing deleterious splicing defects and frameshifts within the coding region (i.e. generally cause qualitative variations) or changes in *cis*-regulatory sequences (i.e. typically cause quantitative variations) (Meyer and Purugganan 2013). In general, strategies based solely on loss-of-function mutations are amenable to short and streamlined breeding cycles in opposition to those requiring gain-of-function variants (Luo *et al.* 2022). Although much of the research regarding domestication has been performed in plants exhibiting a short life cycle (i.e. generally annual, biennial, or perennial grown as annuals) with a focus on the Green Revolution crops (Monroe *et al.* 2020), similar traits (e.g. dwarfism, non-shattering, and increased fruit size) associated with the domestication syndrome can be observed in long-lived perennial fruit crops (Wang *et al.* 2023a). A good example of these drastic evolutionary changes can be found in the domesticated form of apple, *Malus domestica* (Cornille *et al.* 2019). In comparison to its wild ancestor, *Malus sieversii, cultivated apples exhibit a large number of the common domestication features found in the Green Revolution, such as:* (i) modified plant architecture and dwarf phenotype; (ii) increase in fruit size; (iii) improved palatability due to modifications in fruit qualities (e.g. soluble sugars); and (iv) increased fruit quality (e.g. shape, colour, sweetness, and firmness) (Cornille *et al.* 2012, 2019). Similar observations have been made for different pear species (Li *et al.* 2022), peach (Zheng *et al.* 2014; Velasco *et al.* 2016), almond (Velasco *et al.* 2016), apricot (Groppi *et al.* 2021), and many more fruit crops.

**Figure 1.**
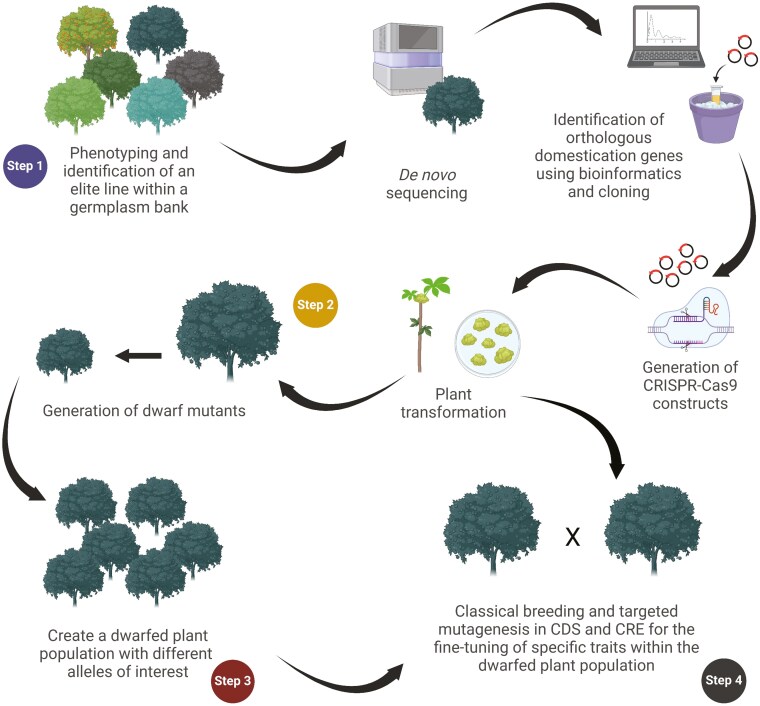
Four-step rapid *de novo* domestication roadmap. (Step 1; Find a candidate species and candidate accessions) The roadmap begins with the identification of an appropriate candidate accession displaying the best agronomical and fruit qualities in a population of wild relatives. Subsequently, this candidate accession will be sequenced to identify orthologous genes involved in the regulation of key traits (e.g. dwarfing). Using different gRNA design software, CRISPR-Cas9 constructs will be generated, and conventional or *in planta* plant transformation performed to knock out the targeted genes. (Step 2; Perform genome editing for a small number of alleles of interest) The first knock-out mutants for these key traits are generated and alteration of the targeted traits is confirmed through thorough phenotypic analysis. In this figure, the dwarfing trait has been used as an example due to its critical use for further breeding purposes. (Step 3; Generate a population with different alleles of interest) The different mutant lines are then pooled to create a population with different alleles of interest to perform classical breeding. (Step 4; Classical breeding for the refined tuning of several domestication alleles) The best mutant lines are used in a classical breeding program to generate elite accessions and fine-tune specific critical agronomical and quality traits with the gradual stacking of loss-of-function variants or variants exhibiting decreased gene expression. Optionally, the offspring generated from these crosses can be further modified using genome editing. To increase the number of breeding cycles, this conventional breeding scheme can use some biotechnological and conventional approaches to accelerate flowering (Fig. 2). CDS, coding sequence. CRE, Cis-regulatory elements.

**Figure 2.**
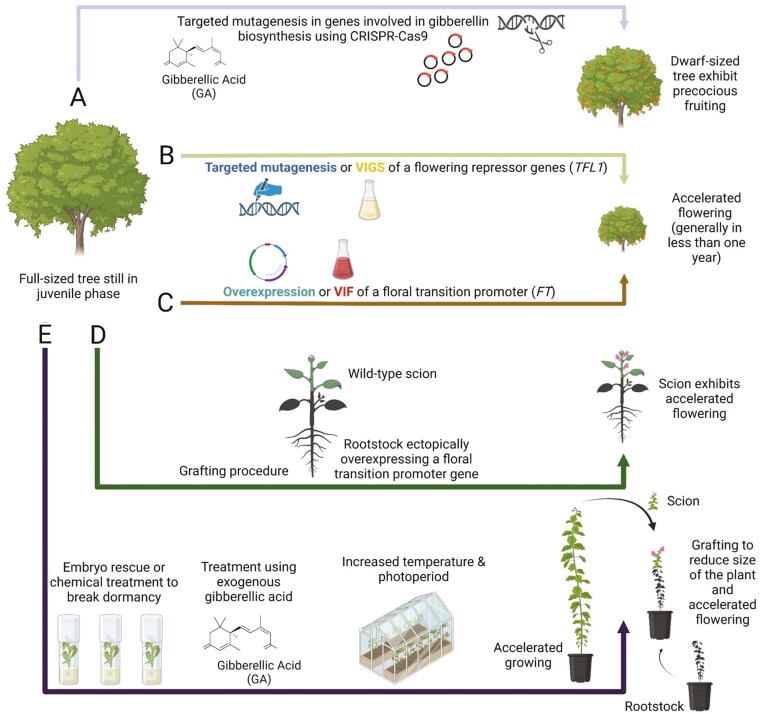
Biotechnological and conventional approaches to hasten flowering in wild relatives. Four biotechnological (A–D) and one conventional (E) strategies are proposed to accelerate fruiting and increase the number of breeding cycles in undomesticated long-lived woody perennials: Dwarfing of genes involved in the synthesis of gibberellic acid using genome editing (A). Rapid cycle breeding using targeted mutagenesis or virus-induced gene silencing through the disruption of a key *FLOWERING LOCUS T* repressor (e.g. *TFL1*) (B). Rapid cycle breeding using overexpression of a floral transition promoter such as *FT* or VIF (C). Acceleration of floral induction in graft-transmitted *FT* (‘Grafting-RBC’) (D). Adaptation of the speed breeding approach (e.g. extended photoperiod, increased temperature, embryo rescue, use of exogenous gibberellic acid, and floral induction through the grafting of speed-bred trees) for large woody perennial crops (E). Biotechnological approaches refer here to methods involving direct genetic modifications (e.g. overexpression of a specific gene) aimed at hastening the flowering process, whereas conventional strategies (e.g. increasing photoperiod and temperature) refer to techniques based on the modifications to the prevailing growing conditions to shorten the plant breeding cycles. Optionally, these techniques are non-exclusive and thus could be possibly combined for an enhanced synergistic acceleration of the flowering phenotype.

The domestication of these major fruit crops started several thousand years ago and was propelled by the introduction of grafting techniques that facilitated the propagation of improved genotypes and the fixation of superior traits (Zohary *et al.* 2012; Cornille *et al.* 2019). As a multifaceted evolutionary process, domestication traits can vary from one fruit crop to another. Fruit size is generally one of the most desirable traits for improvement along with tree size, yield, attractive appearance, and flavour profile (i.e. aroma, polyphenol content, and soluble sugars) (Wang *et al.* 2023a). Several more species-specific traits (e.g. fruit storage, environmental adaptability, and stone size) are generally concomitantly selected with these major domestication traits (Cao *et al.* 2019; Wang *et al.* 2023a). For a rapid crop evolution of underutilized plant species, breeders must rely on a selection scheme based on the positive selection and gradual stacking of loss-of-function mutations in key traits (Fig. 1). The same approach is valid for fruit trees, with the peculiarity that these crops require a step-wise selection process that emphasizes the (i) shortening of the juvenile period and the (ii) modification of their plant architecture right at the beginning of their breeding process as a way to maximize genetic gains and lessen the use of spatial resources. In the case of fruit trees, this is most often achieved by favouring dwarfing traits through the disruption of gibberellins’ (GA) metabolism (Sasaki *et al.* 2002; Bulley *et al.* 2005; Shao *et al.* 2020; Hayat *et al.* 2021) (Fig. 2a). Commercially speaking, dwarfing is one of the most sought-after traits as it can be used to shorten juvenility, increase yield per hectare, facilitate harvest, and ease management procedures (e.g. pruning and pesticide applications) (Atkinson and Else 2001). For rapid domestication, this trait is one of the most useful since crops that already display good fruit quality (i.e. nuts from hickories) can be bred to be more compact and earlier fruiting (Lauri *et al.* 2006; Fazio *et al.* 2014).

## Rapid *de novo* domestication by aiming the right targets

**Figure 3.**
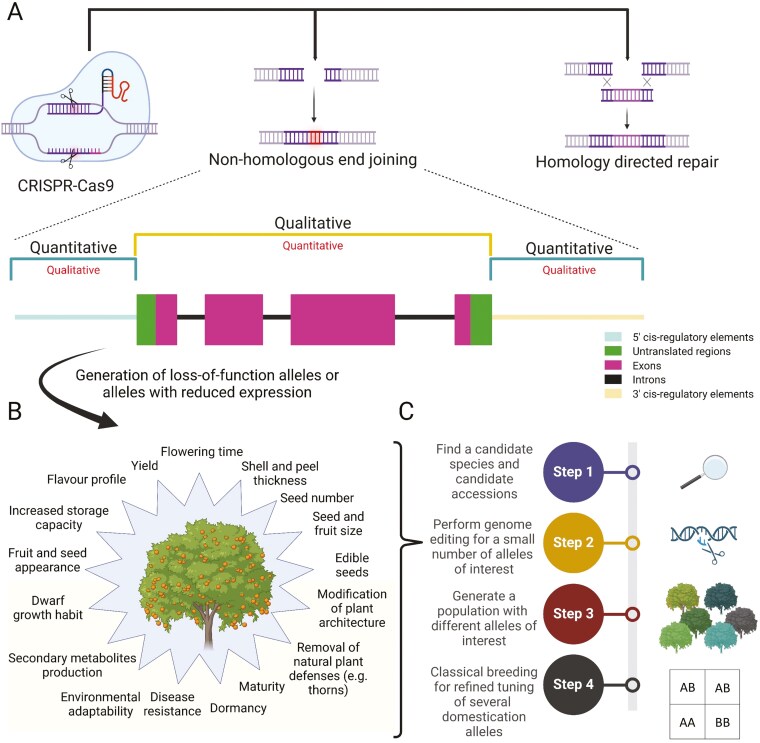
Integration of genome editing in the *de novo* domestication roadmap. (A) Through non-homologous end joining, endonucleases can be used to generate quantitative or qualitative phenotypic modifications by respectively targeting the cis-regulatory elements or coding sequence of a targeted gene. (B) Using genome editing, a plethora of domestication genes can be modified to improve quantitatively and/or qualitatively several agronomical and quality traits. Several examples of rapid *de novo* domestication events for these traits can be found in Table 1. (C) To increase the speed of domestication, genome editing is used at the beginning (i.e. second step) of the proposed rapid *de novo* domestication roadmap (Fig. 1) to rapidly improve several key alleles necessary for further breeding (e.g. dwarfing alleles), agronomical, and/or commercialization purposes. In addition, genome editing can be performed at the fourth step of the pipeline to fine-tune the breeding of specific traits.

At the genomic level, dozens of loci regulate domestication traits which can lead to slow improvement using conventional breeding methods through intended parental crosses (Raina *et al.* 2017). To speed up selection, genome editing is often sought as the holy grail due to its high precision, low cost, high versatility, and ability to generate loss-of-function mutations easily (Fig. 3a). In general, fruit trees and long-lived perennials are more challenging to edit than many annual plants due to the greater complexity of their genetic matrices (e.g. high level of polyploidy and heterozygosity) (Wang *et al.* 2023a). The most prominent genome editing technique currently available is Clustered Regularly Interspaced Short Palindromic Repeats/CRISPR-associated protein (CRISPR-Cas) but other systems, such as Transcription activator-like effector nucleases and Zinc finger nucleases, are also available to scientists (Stella and Montoya 2016). The main components of the Cas9 system are an RNA-guided Cas endonuclease and an sgRNA which both form a Cas ribonucleoprotein cleaving the DNA target (Deltcheva *et al.* 2011; Jinek *et al.* 2012). To perform the cleavage, the protospacer (aka the targeted sequence) needs to be adjacent to a protospacer adjacent motif comprising a motif that can be detected by the Cas protein (Endo *et al.* 2019). After the cleavage, the double-strand breaks (DSB) will be repaired by either of the cell endogenous repair DNA mechanisms which are (i) non-homologous end joining or (ii) homologous-directed repair (HDR) (Pickar-Oliver and Gersbach 2019). Non-homologous end-joining repair is an error-prone pathway in which deletions or insertions may be induced in the specific target site and potentially lead to gene knockouts by disrupting the reading frame (Pickar-Oliver and Gersbach 2019). In the HDR mechanism pathway, cells repair harmful breaks occurring on both DNA strands using an external homologous DNA template which leads to the insertion of this DNA template at the specific target site (Liu *et al.* 2019).

In the literature, there are numerous examples of fast-tracked *de novo* domestication events in annuals or short-lived perennials using genome editing (Table 1). In most cases, targeted mutagenesis has been performed in plants belonging to the Solanaceae (e.g. tomato/*Solanum lycopersicum*, wild tomato/*Solanum pimpinellifolium*, and groundcherry/*Physalis grisea*) (Rodríguez-Leal *et al.* 2017; Lemmon *et al.* 2018; Zsögön *et al.* 2018; Kwon *et al.* 2020; Yuste-Lisbona *et al.* 2020)*, but also other crops such as wild allotetraploid perennial rice (Oryza alta). In S. pimpinellifolium*, *Zsögön *et al.* (2018)*  *used a multiplex CRISPR–Cas9 approach to generate loss-of-function alleles in four domestication genes: (i) LYCOPENE BETA CYCLASE (lycopene synthesis; nutritional quality); (ii) FRUIT WEIGHT (fruit size); (iii) OVATE (fruit shape); and (iv) SELF PRUNING (SP; growth habit and plant architecture). By using this sgRNA-multiplex strategy, the researchers were able to increase the lycopene content by fivefold, fruit size by threefold, and fruit number by tenfold* (Zsögön *et al.* 2018). Similarly, CRISPR-Cas9 modifications have also been performed in groundcherry on agronomically important genes such as *SELF PRUNING*, *CLAVATA1* (fruit size), and *ERECTA* (compactness and plant architecture) (Lemmon *et al.* 2018; Kwon *et al.* 2020). The resulting groundcherry plants from these different experiments exhibited compact growth habits and larger fruit sizes (Lemmon *et al.* 2018; Kwon *et al.* 2020). Similar editing strategies than for groundcherry have also been applied in tomato, a phylogenetically close relative. Kwon *et al.* (2020) developed triple tomato mutants with impaired *SP*, *SP5G*, and *ERECTA* genes that displayed a compact growth habit with an early-yielding phenotype. Furthermore, *Yuste-Lisbona *et al.* (2020)*  *and*  Rodríguez-Leal *et al.* (2017) respectively demonstrated that target mutagenesis in the *EXCESSIVE NUMBER OF FLORAL ORGANS and CLAVATA3-WUSCHEL* genes significantly increases the number of locules and ultimately fruit size in modern tomatoes. In wild *O. alta*, the researchers targeted the homologous genes underlying the *qSH1* (grain shattering; *OaqSH1-CC and OaqSH1-DD*) and *An-1* (awn length; *OaAn-1-CC and OaAn-1-DD*) quantitative trait loci (QTL) as a proof-of-concept for rapid crop evolution in wild crops (Yu *et al.* 2021). By doing so, Yu *et al.* (2021) generated mutants exhibiting non-shattering, due to an absence of a line of abscission between rice grain and the pedicel, and shorter awns.

**Table 1.** Selected publications highlighting the use of CRISPR-Cas9 to edit domestication genes in perennial and annual fruiting crops.

Trait	Species	Gene acronym	Gene name	Effect	Reference
Dormancy Modification of plant architecture	*Actinidia chinensis*	*AcBFT2*	*BROTHER OF FT AND TFL1*	Evergrowing phenotype and increased branching in mutant plants, whereas control lines were exhibiting winter dormancy.	Herath *et al.* (2022)
Dwarf growth habit	*Musa acuminata*	*MaGA20ox2*	*GIBBERELLIN 20 OXIDASE 2*	Significant decrease in plant height of six mutant lines at three developmental stages in comparison to the control lines.	Shao *et al.* (2020)
Flowering time Modification of plant architecture	*Actinidia chinensis*	*AcCEN*	*CENTRORADIALIS*	The mutant lines exhibited compact growth habits with an accelerated terminal flower and fruit development.	Varkonyi-gasic *et al.* (2019)
Fruit and seed appearance	*Solanum lycopersicum*	*SlPSY1*	*PHYTOENE SYNTHASE 1*	Generation of a palette of fruit colours (i.e. from green to dark red).	Yang *et al.* (2023)
Fruit and seed appearance		*SlMYB12*	*MYELOBLASTOSIS* 12		
Fruit and seed appearance		*SlSGR1*	*STAYGREEN 1*		
Fruit size	*Solanum lycopersicum*	*SlENO*	*EXCESSIVE NUMBER OF FLORAL ORGANS*	Increase in fruit size in mutant lines.	Yuste-Lisbona *et al.* (2020)
Increased storage capacity	*Musa acuminata*	*MaACO1*	*AMINOCYCLOPROPANE-1-CARBOXYLATE OXIDASE 1*	An increase in the shelf life of fruits due to delayed ripening associated with a disruption of ethylene production.	Hu *et al.* (2021)
Maturity Yield	*Solanum lycopersicum*	*SlSP*	*SELF PRUNING*	Generation of compact mutant lines that also exhibited an early fruiting process.	Kwon *et al.* (2020)
Maturity Yield		*SlSP5G*	*SELF PRUNING 5G*		
Maturity Yield		*SlSIER*	*ERECTA*		
Modification of plant architecture	*Citrus sinensis*	*CsTAC1*	*TILLER ANGLE CONTROL 1*	Gene-edited mutant lines exhibited a modification in the growth angle of their branches as well as lower auxin and higher cytokinin levels.	Dutt *et al.* (2022)
Removal or introgression of natural plant defenses (e.g. thorns)	*Solanum melongena*	*SmPPO2*	*POLYPHENOL OXIDASE 2*	Mutant lines developed thorny stems.	Kodackattumannil *et al.* (2023)
Shell and peel thickness	*Solanum lycopersicum*	*SlTAGL1*	*TOMATO AGAMOUS-LIKE1*	Creation of mutant lines with a significantly thinner pericarp.	Jeon *et al.* (2024)

So far, several CRISPR systems have been demonstrated to be efficient in a plethora of cultivated fruit crop species, including apple, banana, blueberry, cacao, chestnut, coffee, different citrus species, grapevine, pomegranate, papaya, and pear (Sattar *et al.* 2021; Jacobson *et al.* 2023). In underutilized species, a large breadth of traits can be engineered using Cas9, including plant architecture, height, and fruit quality, to kickstart the domestication process (Fig. 3b and c). In apple, directed mutagenesis was used to induce resistance to the bacterium *Erwinia amylovora*, the causal agent of fire blight disease, by targeting the genomic sequence of *DSPA/E-INTERACTING PROTEINS OF M.9 DOMESTICA 4* (*MdDIPM4*) (Pompili *et al.* 2020). As a result, the researchers successfully generated edited apple trees that exhibited significantly lower rates of necrotic symptoms in comparison to the non-edited wild-type plants (Pompili *et al.* 2020). In banana (*Musa acuminate,* AAA group), genome editing was used to generate semi-dwarf plants by targeting the *GIBBERELLIN 20 OXIDASE 2* (*MaGA20ox2*) gene (Shao *et al.* 2020). In addition, CRISPR-Cas9 was used to generate fruits with an increased shelflife by disrupting *1-AMINOCYCLOPROPANE-1-CARBOXYLIC ACID OXIDASE* (*MaACO1*), a gene involved in the conversion of 1-aminocyclopropane-1-carboxylic acid into ethylene (Hu *et al.* 2021). As a whole, these examples demonstrate the potential of targeted mutagenesis for rapid domestication, but the implementation of gene editing procedures in fruit trees is often challenging due to their recalcitrance to transformation (Table 1).

## Overcoming recalcitrance with *in planta* transformation

**Figure 4.**
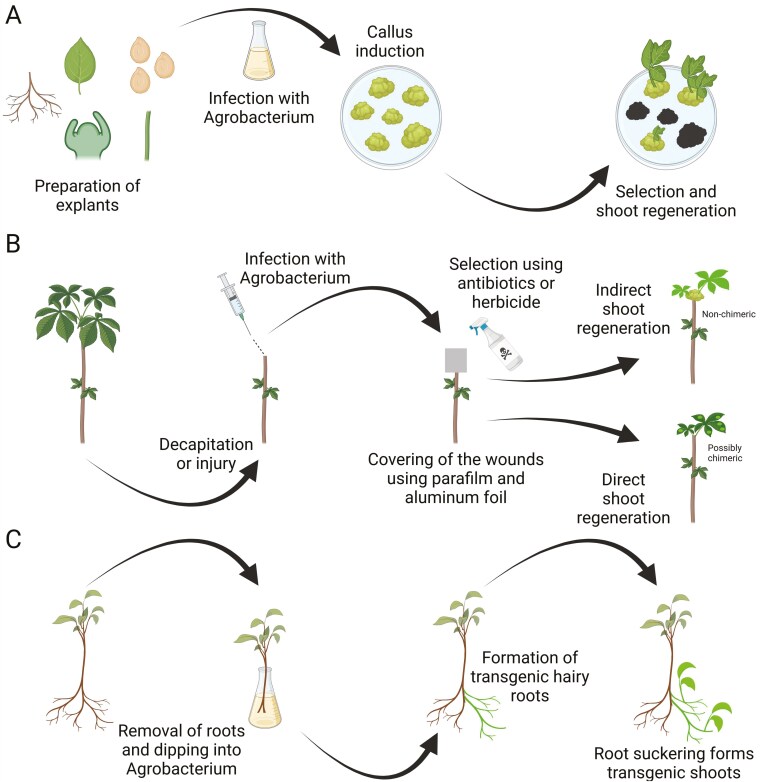
Conventional and in planta transformation techniques of perennial woody plants. (A; Classical transformation and regeneration method) *In vitro* indirect regeneration can be performed using different types of explants (e.g. apical meristems, leaves, stems, seeds, or roots). The method is generally considered slow and inefficient but typically yields non-chimeric plants. (B; *In vivo* direct and indirect regeneration approach) Under this method, the plant is transformed under *in vivo* conditions through wounding and subsequent infection by *Agrobacterium*. From the wound, two pathways (i.e. direct or indirect) can be undergone independently or simultaneously to initiate the regeneration process. In the direct pathway, no intermediate tissue (i.e. callus) is formed, whereas callus is generated in the indirect pathway. (C; Cut-dip-budding approach) This method is performed under *in vivo* conditions and takes advantage of the suckering features of species that can be easily vegetatively propagated. To use the cut-dip-budding strategy, plants with their native roots removed are transformed with *A. rhizogenes*. Subsequently, transgenic roots regenerate from the wound site and subsequently produce shoots due to the innate suckering features of the plant.

Plant stable transformation, the process of inserting exogenous DNA (i.e. the transgene) that is heritable across generations, is a major bottleneck for most crop species, particularly perennial woody plants, and most fruit tree species are considered recalcitrant to *in vitro* callus-based transformation (Fig. 4a). To circumvent recalcitrance to callus regeneration in monocot and dicot species, a large breadth of *in planta* transformation strategies have been developed, with several being suitable for woody perennial species (Gélinas Bélanger *et al.* 2024). The *in planta* concept can be defined as the following: a method of plant genetic transformation featuring no or only minimal tissue culture steps. In this case, minimal is defined by three features: (i) technically simple (i.e. medium composition involves little to no hormones); (ii) a short duration with a limited number of medium transfers; and (iii) regeneration procedure undergone using a differentiated explant that does not undergo a callus development stage. Recently, more than 30 different *in planta* techniques have been characterized which include two [i.e *in vivo* direct and indirect regeneration approach (Fig. 4b) and cut-dip-budding strategy (Fig. 4c)] that exhibit a higher potential for adaptation in a large number of woody perennial species due to their high rates of success, great adaptability, affordable cost, and low requirements in terms of technical requirement (Gélinas Bélanger *et al.* 2024). Both of these techniques rely on *de novo* organogenesis, a phenomenon that occurs upon cell wounding and gives rise to new plant organs via adventitious buds (i.e. direct approach) or callus (i.e. indirect approach) regeneration, to generate transformed shoots (Long *et al.* 2022).

In the *in vivo* callus direct and indirect regenerations strategy, plants are wounded under non-sterile conditions (e.g. lab or field) and transformed using a resuspended solution of *Agrobacterium tumefaciens* cells containing the transgene of interest. Following the wounding phase, the injuries are covered with either parafilm, mud, or aluminium foil, to maintain proper humidity and dark conditions (Zhang *et al.* 2017; Rizwan *et al.* 2021). Subsequently, selection can be undergone using a visual reporter system [e.g. green fluorescent protein (Rizwan *et al.* 2021), β-glucuronidase (Rizwan *et al.* 2021), or RUBY (Zhai *et al.* 2022)], antibiotics [e.g. hygromycin (Zhang *et al.* 2017; Rizwan *et al.* 2021), and kanamycin (Zhang *et al.* 2017)], and herbicides [e.g. phosphinothricin (Zhang *et al.* 2017)]. *In vivo* callus direct (i.e. adventitious bud) and indirect (i.e. callus) regeneration has been demonstrated efficient in a large number of shrubs/trees [e.g. *Populus* spp. (Ma *et al.* 2021; Yuan *et al.* 2022; Nagle *et al.* 2023), *Camellia sinensis* (Zhou *et al.* 2022a), and *Eucalyptus sp.* (Ma *et al.* 2021)], vines [*Passiflora edulis* (Rizwan *et al.* 2021)], fruit trees [e.g. *Citrus sinensis* (Yong *et al.* 2000; Xie *et al.* 2020), *Citrus maxima* (Yong *et al.* 2000; Zhang *et al.* 2017), *Dimocarpus longan* (Yukun *et al.* 2016; Chen *et al.* 2020), *Punica granatum* (Niu *et al.* 2018), and *Ziziphus jujuba* (Shi *et al.* 2015a, b; Shi *et al.* 2016; Wang *et al.* 2017)], perennial cultivated as annuals [e.g. *Solanum lycopersicum* (Pozueta-Romero *et al.* 2001; Mily *et al.* 2020)], and annuals [e.g. *Glycine max* (Li *et al.* 2012; Xia *et al.* 2014; Hu *et al.* 2016), and *Arachis hypogaea* (Zhai *et al.* 2022)].

Recently, the cut-dip-budding was proposed as a novel method to circumvent *in vitro* methods in dicot species with a high suckering capacity such as sweet potato, herbaceous plants (e.g. *Anemone hupehensis*, *Taraxacum kok-saghyz*, and *Coronilla varia*), cane fruits (e.g. *Rubus rosifolius*), and woody species (e.g. *Lycium chinense*, *Broussonetia papyrifera*, and *Aralia elata*) (Lu *et al.* 2023; Cao *et al.* 2023a, 2023b; Cao *et al.* 2024). The cut-dip-budding is similar to the *in vivo* direct and indirect regeneration approach as it relies on the *de novo* regeneration of organs, but differs in that it uses *Agrobacterium rhizogenes* instead of *A. tumefaciens* and targets adventitious buds that will develop into root suckers instead of adventitious shoots. From these root suckers, shoots will eventually emerge and form fully transformed plants. This method is versatile as it can harness the natural suckering features of several wild species to perform transformation in a rapid and reliable manner. Overall, both *in planta* methods presented here can be used to fast-track genome editing and the implementation of rapid cycle breeding procedures in hard-to-transform perennial fruit species.

## Shortening juvenility using rapid cycle breeding and viral vectors

**Figure 5.**
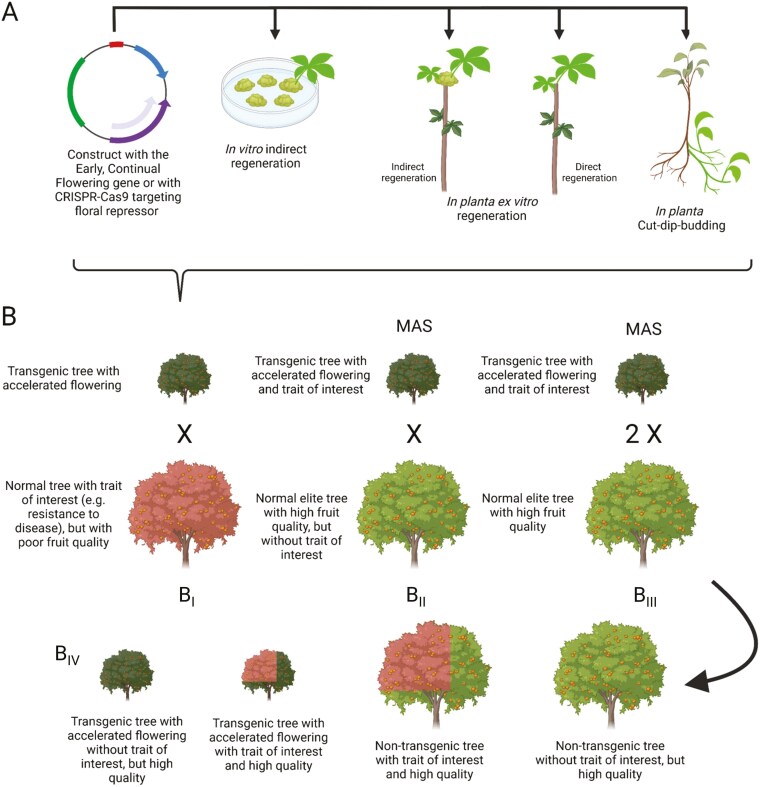
Rapid cycle breeding pipeline to domesticate new crops. (A) Conventional (i.e. indirect *in vitro* regeneration) or *in planta* transformation of a candidate accession with the Early, Continual Flowering trait via the overexpression of *FT* or disruption of *TFL1*. (B) Rapid cycle breeding scheme divided in four main steps. First step is the initial cross between the transgenic early-flowering line with a genotype harbouring a trait of interest (e.g. disease resistance) but with deficient commercial fruit quality (B_I_). Second step is the cross between the transgenic early-flowering line harbouring the trait of interest with a line displaying excellent commercial fruit qualities (B_II_). Third step is the two phases of backcrossing with the original genotype to select the required agronomical and fruit quality traits (B_III_). Fourth step is the selection of the final non-transgenic (i.e. displaying a normal flowering phenotype) genotype exhibiting the trait of interest and commercially acceptable fruit qualities (B_IV_). Steps B_2_ and B_3_ can be performed using MAS to improve the efficiency of the breeding process. The rapid breeding scheme presented in this figure has been inspired by Petri *et al.* (2018).

Dwarfing is one of the most common approaches to shorten juvenility; however, the outcomes of dwarfism on juvenility might not be severe enough for the specific requirements of a breeding program. To circumvent the roadblocks associated with a juvenile phase that might still be too long, several more ‘drastic’ strategies have been developed to trigger flowering at a very young age, often before one year of growth (Flachowsky *et al.* 2009). Interestingly, these methods relying on bioengineering can be combined with *in planta* transformation procedures to increase the speed of the procedure (Fig. 5a).

Rapid cycle breeding, also called ‘FasTrack’ breeding, employs genetic engineering to shorten the juvenile period and hasten flowering (Petri *et al.* 2018; Khan and Korban 2022). In rapid cycle breeding, two approaches can be used to trigger flowering at an early stage of development: (i) downregulate flowering repressors [e.g. *TEMPRANILLO*, *TERMINAL FLOWER1 (TFL1)-like* and *CENTRORADIALIS (CEN)-like* genes] (Varkonyi-gasic *et al.* 2019) (Fig. 2b) or (ii) ectopically express genes involved in floral induction such as *FLOWERING LOCUS T* (*FT*) (Srinivasan *et al.* 2012) (Fig. 2c). In a typical rapid cycle breeding scheme, early flowering mutants are crossed with a cultivar harbouring one or multiple traits of interest (Petri *et al.* 2018) (Fig. 5b). Following the introgression of the loci of interest, backcrosses are performed with the original genotype in combination with marker-assisted selection (MAS) to track the loci of interest (Petri *et al.* 2018). In the last step of the rapid cycle breeding scheme, the *FT* cassette is segregated and offspring without transgenes are selected (Petri *et al.* 2018). In comparison to dwarfing, the whole scheme of a rapid cycle breeding program (i.e. initial cross along with another cross and two additional backcrosses to segregate) takes about 4 years to be completed which is about the same time as for a dwarf tree to reach reproductive maturity (Petri *et al.* 2018).

In kiwifruit, *Actinidia chinensis*, the former approach (i.e. downregulation of flowering repressors) has been used to mutate two genes, *AcCEN4* and *AcCEN*, involved in the repression of the flowering process (Varkonyi-gasic *et al.* 2019). By generating deleterious mutations in these genes, the scientists were able to change the growing and flowering habits of this climbing woody perennial plant with a long vegetative phase (i.e. 5 years) into a compact plant with a rapid flowering (i.e. 9 months) and fruit development. In apple and pear, Charrier *et al.* (2019) created *Mdtfl1.1* and *Pctfl1.1* knockout mutant lines using CRISPR-Cas9 and demonstrated that early flowering was induced in 93% and 9% of these lines, respectively. Similarly, Kotoda *et al.* (2010) demonstrated that *MdTFL1* antisense RNA induces early flowering in apple trees, with some trees displaying their first flowers 8 months after their transfer into the greenhouses. In comparison, the control plants did not flower after more than 6 years of growth. In woodland strawberry (*Fragaria vesca*), RNAi-mediated silencing of *TEMPRANILLO* (i.e. a gene repressing *FT* function) resulted in an earlier flowering phenotype that was sometimes observed even under *in vitro* conditions (Dejahang *et al.* 2023).

For the second rapid breeding cycle approach, extensive research has been conducted with plum trees transformed with the poplar *PtFT1* gene to shorten juvenility (Srinivasan *et al.* 2012). Under greenhouse conditions, the plum trees overexpressing this gene hastened fruit production, with fruiting starting from 1 to 10 months of age, and also displayed a continuous production of fruits (Srinivasan *et al.* 2012). A similar strategy has been successfully used by Tränkner *et al.* (2010) to trigger early flowering in poplar and apple using an *MdFT* overexpression cassette driven by the CaMV 35S. In Tränkner *et al.* (2010), the use of this cassette resulted in the creation of a transgenic apple line, named ‘T780’, which exhibited flowering during *in vitro* cultivation. In addition to this line of evidence, Kotoda *et al.* (2010) also demonstrated that overexpression of *MdFT1* in transgenic apple lines results in early flowering. The trifoliate orange (*Poncirus trifoliata*) is a good candidate for domestication due to its high resistance to cold, fungus *Phytophthora parasitica* (root rot), and the tristeza virus (Khan *et al.* 2021). Rapid cycle breeding has been successfully performed in this species through the ectopic use of an overexpression cassette containing the citrus *FT* (*CiFT*) gene which hastened flowering (Endo *et al.* 2005). Similar results were obtained with the ‘Carrizo’ citrange (*Citrus sinensis* × *Poncirus trifoliata*) plants that were constitutively expressing the floral identity genes *LEAFY* (*LFY*) or *APETALA1* (*AP1*) from *Arabidopsis*, with the induction of flowering 12–20 months after their transfer to greenhouse conditions (Peña *et al.* 2001). Although this system has been proven efficient in several plant species, some studies have encountered roadblocks. For example, the overexpression of the citrus *FT3* gene in several citrus species (‘Carrizo’ citrange, ‘Hamlin’ sweet orange, and ‘Duncan’ grapefruit) led to precocious flowering in Petri plates which could not be converted to shoots (Moore *et al.* 2016). Recently, a novel system using a heterologous *FT* (*FTa1*) gene under the control of an alcohol-inducible promoter has been demonstrated to be efficient at inducing customizable flowering in *Arabidopsis* (Yeoh *et al.* 2011). Upon exposure to ethanol, *Arabidopsis* plants modified with *FTa1* rapidly demonstrated synchronous flowering (Yeoh *et al.* 2011). The method still needs to be validated in fruit trees but could be potentially harnessed to customize and schedule the floral induction of parental lines (Yeoh *et al.* 2011).

VIF is a valid alternative for plants that are recalcitrant to conventional or *in planta Agrobacterium* transformation (McGarry *et al.* 2017; Maeda *et al.* 2020). To do so, wood perennial plants are inoculated with a viral vector performing the silencing (VIGS) of a florigen repressor (e.g. *TFL1*) (Fig. 2d), a viral vector expressing a floral initiator (e.g. florigen, *FT* gene) (Velázquez *et al.* 2016; Maeda *et al.* 2020) (Fig. 2e), or both at the same time (Yamagishi *et al.* 2014). The VIF approach has been previously used to accelerate the flowering of apple (Sasaki *et al.* 2011; Yamagishi *et al.* 2014) and grapevine (Maeda *et al.* 2020) seedlings using the *Apple latent spherical virus*. In addition, the technique has been used to overcome juvenility in citrus plants using *Citrus leaf blotch virus* (Velázquez *et al.* 2016). Although more research needs to be undertaken to find appropriate viral vectors for a large number of understudied species, this technique is an interesting alternative to accelerate the flowering and increase the number of breeding cycles of wild plants.

## Inducing flowering with grafting and growing conditions

Grafting involves the cutting and joining of different plant tissues together, often from different cultivars or species, to ensure their fusion through the regeneration of a callus mass (Feng *et al.* 2024). The technique of grafting has been practiced for over 2500 years and is now a mainstay in the commercial propagation of a large number of ornamental and fruiting plants (Melnyk and Meyerowitz 2015). To shorten the juvenile period, different grafting techniques have been developed (Fig. 2f).

The first of these techniques, herein called ‘Grafting-RBC’, aims at inducing flowering to a scion using a rootstock that has been transformed using an *FT* cassette. Naturally, *FT* sequences move from leaf tissues to the shoot apical meristems upon transcription (Jackson and Hong 2012). In *Jatropha curcas*, this natural phenomenon has been harnessed to induce rapid flowering by grafting wild-type scions onto *FT* overexpressing rootstocks (Tang *et al.* 2022). As a graft-transmissible mobile florigen, the grafting of wild-type scions onto *FT* overexpressing rootstocks triggers extreme precocious flowering in *J. curcas in vitro* clones (Tang *et al.* 2022). As these flowering clones failed to produce roots, the researchers developed a rescue method in which shoots with flower buds were cloned on wild-type seedling rootstocks (Tang *et al.* 2022). Interestingly, researchers have demonstrated that this approach can be used to induce flowering in other plant species of the *Jatropha* genus (e.g. *J. gossypifolia, J. integerrima, J. multifida,* and *J. podagrica*) using interspecific grafting (Tang *et al.* 2022). Similar results have been achieved by grafting regular northern highbush blueberry (*Vaccinium corymbosum*) onto plants overexpressing *VcFT* (Song *et al.* 2019), thus demonstrating that the method can be used to trigger early flowering in northern shrubs. In Notaguchi *et al.* (2020), a large number of distantly related plant species have been demonstrated to be graft-compatible to some extent, thus suggesting that the ‘Grafting-RBC’ technique could be performed by grafting scions of hard-to-transform species onto the rootstocks of easy-to-transform model plants such *A. thaliana* and *Nicotiana sp.*.

Another approach to trigger early flowering is to manipulate growing conditions (Nocker and Gardiner 2014) (Fig. 2g). In long-lived woody perennials, the length of the juvenile period is influenced by the prevailing environmental conditions (Hackett 2011; Nocker and Gardiner 2014). Stressful conditions that are deleterious to vigor (e.g. defoliation, cold and water stresses, low light, and mineral deficiencies) generally postpone the transition to the adult phase, while conditions favouring an increase in vigor tend to do the opposite (Hackett 2011). In apple, a crop with a generation time of about 5–8 years from seedling to the adult phase, Aldwinckle (2022) developed a method to induce flowering in 16–20 months (and sometimes in less than 10 months) after germination. To do so, plants are grown continuously under conditions conducive to rapid growth (i.e. high temperature, long photoperiod, heavy fertilization, and continuous plant control) in a similar fashion to what is being practiced in the speed breeding of field crops (Watson *et al.* 2018; Aldwinckle 2022). Subsequently, the mature apical portion of the speed-cultivated plant can be grafted onto a rootstock for ease of maintenance and further growth (Nocker and Gardiner 2014). Additional experiments in apple and crabapples (*M. hupehensis*) have been proven successful in the past and thus need to be reconsidered for the domestication of new species in the wake of the expansion of speed breeding in field crops (Karnatz 1970; Visser 1970; Zimmerman 1971). To speed up the breeding process, seeds can be treated with exogenous applications of GA or embryo rescue to overcome dormancy and circumvent the time-consuming vernalization process (Nocker and Gardiner 2014). Overall, just like speed breeding, this approach demonstrates a lot of potential for fruit crops with a small to moderate size (e.g. naturally or engineered dwarfed plants).

## Candidate plant species for *de novo* human-driven crop evolution

In high latitudinal regions, candidate fruit and nut crop species for domestication abound, but the information regarding them is often circumscribed to the grey literature (e.g. reports from nurseries, governments, and local scientific societies). Moreover, genomic data that could be harnessed for their breeding is normally absent or (in the best cases) very limited. In the last 20 years, cultivars from many novel fruit crops, such as haskap (*Lonicera caerulea*) (Thompson and Barney 2007; Cheng *et al.* 2023), sea buckthorn (*Hippophae rhamnoides*) (Kalia *et al.* 2011; Ruan *et al.* 2013; Nybom *et al.* 2023), hardy kiwi (*Actinidia arguta*) (Cossio *et al.* 2014; Latocha *et al.* 2021), arctic kiwi (*Actinidia kolomikta*) (Paulauskienė *et al.* 2009; Pranckietis *et al.* 2009), dwarf sour cherries (*Prunus fruticose* ×* P. cerasus*) (Bors 2001; Bors and Sawatzky 2007), saskatoon berries (*Amelanchier alnifolia*) (Żurawicz *et al.* 2012; Bieniek *et al.* 2019), and aronia (*Aronia melanocarpa*) (Jeppsson 1996; Brand 2010, 2013), have been bred by academic institutions and governmental research agencies and are now available for propagation. The short juvenility, about 3 to 5 years, small stature, and exceptional hardiness of the aforementioned species probably contributed to their breeding success and commercial spread. Still, the genetic improvement status of a large number of species, including vines (e.g. *Passiflora incarnata*), shrubs (e.g. *Chaenomeles* spp., *Viburnum* spp., and *Sheperdia argentea*), fruit trees (e.g. *Prunus mandshurica*, and *Mespilus germanica*) and several nut trees (e.g. *Carya* spp., *Juglans* spp., *Corylus* spp., and *Staphylea* spp.), remains largely stagnant. To a great extent, the fruits of these northern perennials are gathered in the wild or cultivated in local small-scale farms by passioned *aficionados* and most of their genetic improvement has been undergone through dedicated local nurseries and local organizations aiming to widespread their use. On the whole, Table 2 provides an overview of 16 candidate uncultivated species hardy to United States Department of Agriculture (USDA) plant hardiness zone 6 or less with a potential for rapid breeding and specific information regarding their taxonomy, domestication status, benefits, and limitations.

**Table 2.** Selected cold-hardy species for *de novo* domestication.

Family	Genus	Species	Common name	Domestication and cultivation status	Size	Description of fruits and/or seeds	Hardiness (USDA zone)	Benefits	Limitations	Proposed modifications	Reference
Adoxaceae	*Viburnum*	*lentago*	Nannyberry	Undomesticated. Small-scale cultivation.	Moderate shrub (3–6 m)	Berry-like drupes with a blue-black colour.	2	Extremely hardy. Fruits exhibit good taste.	Small size of the fruits. Plants could be smaller to facilitate mechanical harvest.	Dwarfing. Increase fruit size. Modify the architecture of the plant to facilitate harvesting.	Spriggs (2019); Missouri Botanical Garden (2024); Plants for a Future (PFAF). (2019)
Adoxaceae	*Viburnum*	*opulus*	American cranberrybush, highbush cranberry	Undomesticated. Small-scale cultivation.	Small to moderate shrub (2.5–3.5 m)	Small cranberry-like red berries.	2	Extremely hardy. Fruits exhibit good taste when cooked.	Small size of the fruits. Plants could be smaller to facilitate mechanical harvest.	Dwarfing. Increase fruit size. Modify the architecture of the plant to facilitate harvesting.	Kollmann and Grubb (2002); Missouri Botanical Garden (2024); Plants for a Future (PFAF). (2019)
Ginkgoaceae	*Ginkgo*	*biloba*	Maidenhair tree	Domesticated. Cultivated mainly in Asia.	Large tree (25–30 m)	Fruit-like cones contain edible seeds characterized by a foul scent.	3	Fruits are widely consumed in Asia.	Large tree with a long juvenile stage. The fruits contain ginkgotoxin, a neurotoxin.	Dwarfing. Eliminate potential adverse effects associated with ginkgotoxin by disrupting its biosynthesis pathway.	Gu *et al.* (2017); Missouri Botanical Garden (2024); Plants for a Future (PFAF). (2019)
Juglandaceae	*Carya*	*ovata*	Shagbark hickory	A few named cultivars were selected from the wild and are available in a few nurseries. Uncultivated for consumption.	Large tree (30 m)	Pecan-like nuts encased by a large hard shell.	4	Nuts exhibit a good taste and large in size.	Large trees with a long juvenile stage. Hard-to-crack shell which can be a limiting factor for commercialization.	Dwarfing. Breed cultivars with thinner shells.	Graney (1990); Plants for a Future (PFAF). (2019)
Juglandaceae	*Juglans*	*nigra*	Black walnut	A few named cultivars were selected from the wild and are available in a few nurseries. Small-scale cultivation.	Large tree (20–40 m)	A walnut-like seed protected by a hard-to-crack shell	4	Seed size is quite large. Very hardy.	Large trees with a long juvenile stage. Hard-to-crack shell which can be a limiting factor for commercialization.	Dwarfing. Breed cultivars with thinner shells.	Williams (1990); Missouri Botanical Garden (2024); Plants for a Future (PFAF). (2019)
Juglandaceae	*Juglans*	*cinerea*	Butternut	A few named cultivars were selected from the wild and are available in a few nurseries. Small-scale cultivation.	Large tree (30 m)	A walnut-like seed protected by a hard-to-crack shell.	3	Seed size is quite large. Very hardy.	Large trees with a long juvenile stage. Hard-to-crack shell which can be a limiting factor for commercialization.	Dwarfing. Improve the crackability of the shell. Improve resistance to canker fungal disease.	Rink (1990); Missouri Botanical Garden (2024); Plants for a Future (PFAF). (2019)
Rosaceae	*Amelanchier*	*stolonifera*	Running serviceberry, Quebec Berry	Undomesticated. Small-scale cultivation.	Small shrub (1–1.5 m)	Small blueberry-like edible berries that are dark-purple.	4	Extremely hardy. Small size of the plant facilitates mechanical harvesting.	Small berries. Suckering growth habit.	Increase fruit size and reduce suckering.	Stushnoff (1991); Missouri Botanical Garden (2024); Plants for a Future (PFAF). (2019)
Rosaceae	*Chaenomeles*	*speciosa*	Flowering quince	A few named cultivars mainly for ornamental purposes. Cultivated mainly for ornamental purposes.	Small to moderate shrub (2–3 m)	Quince-like edible golden-yellow fruits.	4	Small hardy shrub. Fruits are a good size but require cooking.	Suckering habit. Fruits are hardly palatable when eaten raw.	Improve the palability and flavour profile of the fruits. Improve productivity. Limit suckering growth habit.	Rumpunen (2002); Plants for a Future (PFAF). (2019)
Rosaceae	*Prunus*	*maritima*	Beach plum	A few named cultivars were selected from the wild and are available in a few nurseries. Small-scale cultivation.	Small to moderate shrub (2–2.5 m)	A small plum with a sub-acid to sweet taste.	3	Shrub is quite adaptable and tolerates maritime conditions.	Small fruits. Suckering shrub.	Modify plant architecture to improve mechanical harvesting. Increase fruit size. Limit suckering growth habit.	Uva (2003); Uva and Whitlow (2007); Plants for a Future (PFAF). (2019)
Rosaceae	*Sorbus*	*americana*	Rowanberries, mountain ash	A few named cultivars available mainly for ornamental purposes. Cultivated mainly for ornamental purposes.	Small tree (5–10 m)	Clusters of bitter-tasting orange-red drupes.	3	Extremely hardy and dependable.	Fruits contain high levels of tannin causing the fruits to be bitter.	Dwarfing. Reduce levels of tannin to decrease the bitterness of the fruits. Increase fruit size.	Missouri Botanical Garden (2024); Plants for a Future (PFAF). (2019)
Rutaceae	*Poncirus*	*trifoliata*	Trifoliate orange	A few named cultivars. Used mainly as a rootstock for different citrus species or for interspecific breeding.	Moderate shrub (3–5 m)	Acidic and bitter citrus-like fruits.	5/6	Hardiest citrus. Used as rootstock to increase hardiness. Highly productive	Fruits are seedy and unpalatable when consumed fresh. Trees are extremely thorny.	Improve flavour profile. Generate seedless triploids. Improve hardiness.	Nesom (2014); Missouri Botanical Garden (2024); Plants for a Future (PFAF). (2019)
Sapindaceae	*Aesculus*	*hippocastanum*	Horse chestnut	Undomesticated. Relatively common as an ornamental plant.	Large tree (20–25 m)	The fruit is a globular dehiscent capsule containing 1–2 seeds.	3	Large size of the seed. Very hardy.	Large tree with a long juvenile stage. Aesculin and aescin (i.e. a form of saponin) are toxic compounds present in the seed	Dwarfing. Disrupt the synthesis of toxic compounds.	Gastaldo *et al.* (1994); Missouri Botanical Garden (2024); Plants for a Future (PFAF). (2019)
Sapindaceae	*Xanthoceras*	*sorbifolium*	Yellowhorn	Domesticated. Cultivated mainly in Asia.	Small tree (5–8 m)	Fruits are capsules that contain 6–18 small black edible seeds. The fruits resemble small buckeyes.	4	Drought and cold-tolerant. Taste of the seeds is similar to macadamia nuts. Several cultivars have been developed in China.	Inflorescence fertility is a major issue.	Dwarfing. Improve inflorescence fertility using the all-female-flowers trait.	J Zhou *et al.* (2022b); X Wang *et al.* (2023b); Plants for a Future (PFAF). (2019)
Staphyleaceae	*Staphylea*	*trifolia*	American bladdernut	Undomesticated. Small-scale cultivation.	Moderate shrub (2–4 m)	Bladder-like seed capsules	4	Interesting size for production. Highly productive. Hardy.	Small size of the seed. Challenge in separating the nut from the shell.	Increase seed size. Improve plant architecture to facilitate harvest.	Spongberg (1971); Garwood and Horvitz (1985); Missouri Botanical Garden (2024); Plants for a Future (PFAF). (2019)

## Conclusion

Sought as a long evolutionary process for thousands of years, domestication can now be short-cut using novel biotechnologies. For orphan crops, this means that breeding programs can now fast-track their development using a genome-editable catalog of traits. In high latitudinal regions, many plant species have the potential to become new rising stars if given the time and resources required for such an endeavour. Importantly, the diversification of the crop portfolio in northern areas can catalyse an increase in our resilience towards changing climates through a reduction of our overreliance on a small set of crop species grown in southern regions. Largely, this review focussed on genetic improvement technologies that can bolster a rapid and precise human-driven crop evolution. Still, many strategies, such as chemical mutagenesis (e.g. ethyl methanesulfonate and colchicine) (Oladosu *et al.* 2016), microbiome-oriented breeding (Oladosu *et al.* 2016), wide hybridization (Anushma *et al.* 2021), and genomics-assisted breeding (e.g. genomic selection and MAS) (Migicovsky and Myles 2017), also exhibit tremendous potential for accelerated crop evolution and thus need to be considered. Although the domestication toolbox is expanding for fruit trees, numerous challenges still hinder our capability to foster these projects, including: (i) a lack of financial support and infrastructures to pursue these endeavours; (ii) a shortage of genomic data and cloning tools (e.g. promoters) to support efficient genome editing; and (iii) a scientific culture triggered towards fast-science that can generate rapidly publishable results (Leite and Diele-Viegas 2021). Overall, the tools described in the present manuscript aim to offset some of these challenges and provide credible technological solutions to kick-off and achieve these long-term domestication projects. Deemed unrealistic a decade ago, the possibility of generating fruitful breeding projects with orphan crops is becoming more and more tangible as the cost of technology continues to decrease and discoveries are made.

## Data Availability

No new data were generated or analysed in support of this research.
